# Association between Pediatric Adenovirus Infection and Type 1 Diabetes

**DOI:** 10.3390/children9101494

**Published:** 2022-09-29

**Authors:** Amany A. Arafa, Adel Abdel-Moneim, Rehab G. Khalil, Waled M. El-Senousy, Mahmoud M. Kamel, Dalia Y. Kadry, Gamal Allam, Ahmed S. Abdel-Moneim

**Affiliations:** 1 Molecular Physiology Division, Faculty of Science, Beni-Suef University, Beni-Suef 62511, Egypt; 2 Immunology Division, Faculty of Science, Beni-Suef University, Beni-Suef 62511, Egypt; 3 Department of Water Pollution Research, Environmental Research Division, National Research Centre (NRC), Dokki, Cairo 12622, Egypt; 4 Department of Clinical Pathology, National Cancer Institute, Cairo University, Cairo 11796, Egypt; 5 Department of Microbiology, National Cancer Institute, Cairo University, Cairo 11796, Egypt; 6 Microbiology Department, Virology Division, College of Medicine, Taif University, Taif 21944, Saudi Arabia

**Keywords:** type 1 diabetes, adenoviruses, cytokines, lipid profile, correlation

## Abstract

Background: Viruses are among the inducers of type 1 diabetes (T1D) as they are implicated in the initiation of β-cell destruction. This study aimed to explore the link between adenoviruses’ infection, inflammatory biomarkers, and the development of T1D. Methods: The study population included 80 children with T1D and 40 healthy controls (2–16 years old). The T1D group was further clustered into two groups according to time of T1D diagnosis: a group of children who were diagnosed during the first year of life and a second group who were diagnosed after the first year of life. Adenovirus DNA, anti-adenovirus IgG, cytokines, and lipid profiles were screened in the different groups. The results were statistically assessed using one-way analysis of variance (ANOVA) and LSD *t*-test. Results: Positive adenovirus PCR was detected in 2.5% and 20% of normal and T1D children, respectively. Moreover, the positive PCR results for adenovirus were found significantly higher in the T1D group, who were diagnosed during the first year of life (33.4%), in comparison to those diagnosed after the first year of life (12%). Anti-adenoviruses IgG was found in 12.5% and 40% of healthy controls and diabetic children, respectively. Seropositive results were found to be higher in newly diagnosed children (46.7%) in comparison to those previously diagnosed with T1D (36%). Body mass index (BMI), IFN-γ, IL-15, adiponectin, lipid profile, and microalbuminuria were significantly increased in T1D adenoviruses-positive children compared to children who were negative for adenoviruses. Conclusions: Adenovirus infection could be among the contributing risk factors and may play a role in the induction of T1D in children.

## 1. Introduction

Type 1 diabetes (T1D) is a multifactorial autoimmune disease that is marked by a depletion of the β cells in the pancreas. Both high hyperlipidemia and dyslipidemia have been reported in T1D patients [1]. A potential relationship between viruses and T1D has been suggested, with many viruses being incriminated, including enteroviruses, rotavirus, cytomegalovirus, Epstein–Barr virus, parechovirus, influenza, parvovirus, mumps, rubella [2].

Human adenoviruses (HAdVs) are the causative agents of several clinical syndromes, including gastroenteritis, respiratory disease, and conjunctivitis [3]. Importantly, HAdV-C was associated with islet autoimmunity [4]. In early life, infection with HAdV-C results in respiratory affections with a lower risk of developing an autoimmune disorder. It remains to be investigated whether having adenovirus C in early life protects one from developing beta-cell autoimmunity. Adenoviruses use the same beta-cell surface receptor as Coxsackievirus B, which may offer a clue to explaining this connection [5]. Conflicting results were found regarding the role of HAdV in the induction of diabetes. Although one study reported a low incidence of T1D diabetes in AdV36 seropositive patients [6], another study revealed that a higher incidence of T1D is correlated with previous infection with AdV36 [7].

The mechanisms leading to the disease involve interactions between the virus, the pancreas, and the immune system. Immunologic and metabolic biomarkers are widely used in the diagnosis of T1D or β-cell destruction [8]. Interferon (IFN)-γ is a crucial cytokine in various immune responses produced by multiple cell types [9] and has long been considered a contributor to autoimmune T1D. Interleukin-15 (IL-15) is a proinflammatory cytokine that promotes the activation and maintenance of natural killer and CD8 (+) memory T cells and has a critical role in the pathogenesis of autoimmune diabetes [10]. Adiponectin, a secretory protein and the most plentiful adipokine in circulation, induces insulin sensitivity and exhibits anti-inflammation and antiatherogenic effects [11]. β-cell dysfunction and apoptosis were found to be linked with alteration in the cytokine profile in pancreatic islet and plasma. Cytokines, such as tumor necrosis factor (TNF)-α, IFN-γ, leptin, and adiponectin, were found to be responsible for the regulation of pancreatic β-cell function [12].

The association between adenovirus infection, inflammatory status, and T1D has not previously been addressed. Therefore, the current study aimed to explore the link between the adenovirus infection, inflammatory biomarkers, and the development of T1D.

## 2. Materials and Methods

### 2.1. Study Population

The study population consisted of 80 children with T1D and 40 healthy controls. These children were treated in the Governmental Hospital, Beni-Suef, Egypt from October 2018 to January 2019. Patients were diagnosed as having T1DM according to the WHO criteria. The healthy controls were 3–15 years old. Children with T1D were divided into two groups: newly diagnosed T1D and previously diagnosed T1D patients according to the duration of diabetes. Newly diagnosed T1D patients were children diagnosed with T1D in the previous year; those who had been diagnosed for more than one year were considered as previously diagnosed T1D patients. The newly diagnosed T1D group contained 30 children ranging 2–16 years old, while previously diagnosed T1D patients group contained 50 children ranging 4–16 years old. All participants were free from infectious diseases and neoplastic, inflammatory, and autoimmune disorders. Patients receiving immunomodulatory drugs were excluded from being enrolled in the study. All T1D patients were treated with insulin (0.5 to 1.5 U/kg body weight/day based on the HbA1c value).

### 2.2. Biochemical Assays

Glycosylated hemoglobin (HbA1c) assays were performed using a MyBioSource kit (CA, USA). C-peptide ELISA kit was purchased from DRG Diagnostics (Marburg, Germany). The colorimetric enzymatic method was adopted to detect the levels of fasting blood sugar (FBS), creatinine, triglyceride (TG), total cholesterol (TC), and high-density lipoprotein cholesterol (HDL-c) using Spinreact kits (Girona, Spain). Low-density lipoprotein cholesterol (LDL-c) levels were estimated using the formula of Friendewald et al. [13]. Microalbuminuria in the urine was estimated using a MedicalExpo kit (Marseille, France), following the manufacturer’s protocol.

### 2.3. Viral DNA Extraction

Total DNA was extracted as previously described [14]. Briefly, a serum sample (70 µL) was added to 50 µL of 1X PCR buffer containing 0.2 mg of proteinase K/mL. After mixing, the sample was incubated at 37 °C for 1h, and then the suspension was boiled for 10 min and centrifuged at 12,500 rpm for 5 min at 4 °C. The supernatant was collected and stored at −20 °C until being used in the PCR.

### 2.4. Nested PCR for Human Adenoviruses

The PCR amplification was conducted using the Ad40/hexAA 18858–18883 5′-GCCGCAGTGGTCTTACATGCACATC-3′, Ad41 (hexon) 19136–19158 hexAA1913, 5′-CAGCACGCCGCGGATGTCAAAGT-3′, for the first run and Ad2 (hexon) 18937–18960 Nested nehexAA18935′-GCCACCGAGACGTACTTCAGCCTG-3′ and Ad2 (hexon) 19051–19079 Nested nehexAA1905 5′-TTGTACGAGTACGCGGTATCCTCGCGGTC-3′ for the nested as previously described [15]. PCR amplification was conducted in a 50 µL reaction mixture containing 10× buffer (10 mM Tris-HCl [pH 9.0], 50 mM KCl, 1.5 mM MgCl_2,_ 0.01% gelatin (wt/vol), 0.1% triton X-100, 200 µm dNTPs, 0.5 µm for each primer, and 2U of Taq DNA polymerase (Thermo). The cycling condition included an initial denaturation step for 4 min at 94 °C. The conditions for amplification consisted of denaturing at 92 °C for 90 s, 55 °C for 90 s, and 72 °C for 120 s. This was followed by a second new PCR run using nested primers. PCR products (10 µL) were analyzed by electrophoresis on 3% agarose gels (Panreac-Spain) and DNA ladder (Biolabs, England) with a size range of 72–1353 bp.

### 2.5. Determination of Anti-Adeno IgG

An enzyme-linked immunoassay (ELISA) classic adenoviruses IgG kit (Serion GmbH, Würzburg, Germany) was used to screen the anti-adenoviruses according to the kit instructions.

### 2.6. Detection of IFN-γ, IL-15, and Adiponectin

Serum levels of IFN-γ, IL-15, and adiponectin were detected using Sandwich ELISA kits from R&D Systems (Minneapolis, MN, USA). All procedures were conducted according to the manufacturer’s procedure.

### 2.7. Statistical Analysis

Statistical analysis was conducted using SPSS version 20 (SPSS Inc., Chicago, IL, USA). Results were expressed as presented as mean ± SD. One-way analysis of variance (ANOVA) and LSD *t*-test were used to compare the results of different groups. Different items in the newly diagnosed T1D, previously diagnosed T1D, and control groups were correlated together using bivariate Spearman correlation to determine whether there were possible links between different parameters with regard to exposure to adenovirus infection.

## 3. Results

### 3.1. Demographic Data

**Figure 1:**
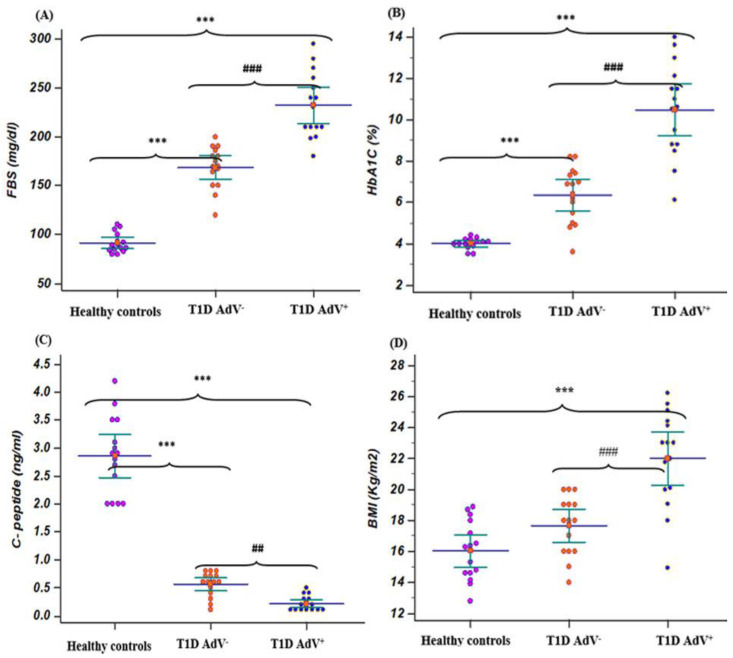
Fasting blood sugar (FBS), hemoglobin A1C (HbA1c), C- peptide, and body mass index in T1D—negative AdV (PCR and antibodies) and T1D—AdV positive (PCR) groups in comparison to healthy control. (**A**) FBS, control group: mean 91.4 ± 4.4 and range 80–110; T1D AdV^−^: mean 168.7 ± 10.65 and range 120–200; and T1D AdV^+^: mean 232.2 ± 16.78 and range 180–295; (**B**) HbA1C, control group: mean 4 ± 0.25 and range 3.5–4.4; T1D AdV^−^: mean 6.36 ± 0.68 and range 3.6–8.2; and T1D AdV^+^: mean 10.46 ± 1.13 and range 6.1–14; (**C**) C-peptide, control group: mean 3.91 ± 0.48 and range 2.7–5.6; T1D AdV^−^: mean 0.56 ± 0.219 and range 0.1–0.8; and T1D AdV^+^: mean 0.213 ± 0.135 and range 0.1-0.5; and (**D**) BMI, control group: mean 17.3 ± 2.2 and range 12.8–30; T1D AdV^−^: mean 16.46 ± 1.15 and range 13–20; and T1D AdV^+^: mean 22 ± 1.55 and range 14.9–26.2. Values were considered significantly different at *** *p* < 0.001 versus healthy controls, and ### *p* < 0.001 T1D—AdV-versus T1D—AdV PCR + children.

Age showed significant differences (*p* < 0.001) among the previously diagnosed T1D group. In children aged 11–15, Mo showed the highest incidence of T1D: 39/50 (78%) and 13/30 (43%) for the previously diagnosed T1D and recently diagnosed T1D groups, respectively (Table 1). Moreover, gender did not show significant differences in terms of the incidence of T1D in the different groups. Similarly, the T1D—AdV^−^ and T1D—AdV^+^ (antibodies or PCR) groups showed a nonsignificant difference among different age groups, gender, and BMI (Table 1). In contrast, the BMI value was significantly higher in the T1D—AdV^+^ group compared to the T1D—AdV- group when comparing the 17 PCR positive patients with matched numbers of uninfected T1D patients (AdV seronegative/AdV PCR negative) and the control nondiabetic group (*p* < 0.001; Figure 1).

**Figure 2:**
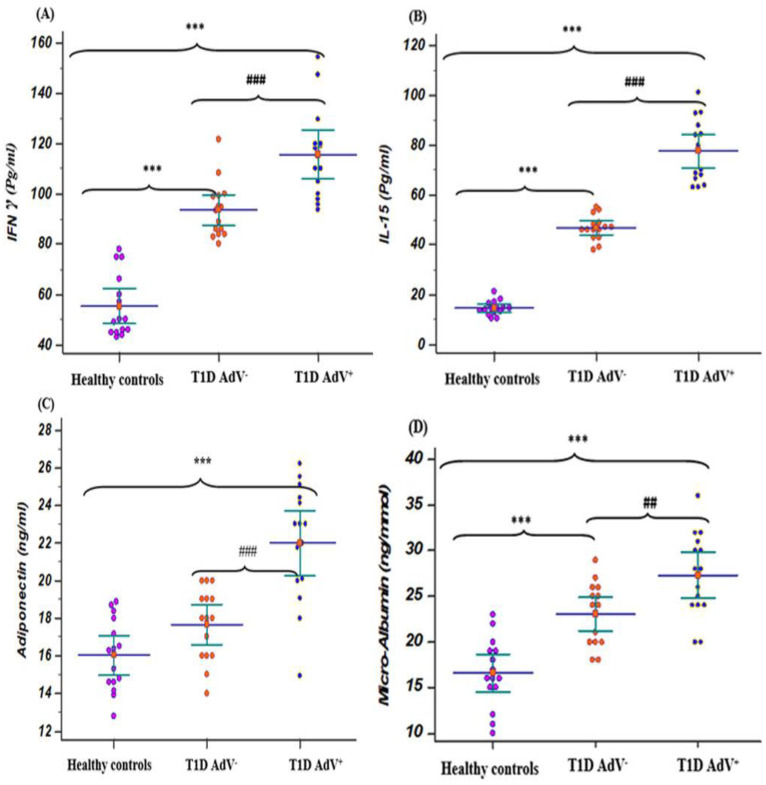
Levels of IFN **γ**, IL-15, adiponectin, and microalbuminuria in the T1D—negative AdV (PCR and antibodies) and T1D—AdV positive (PCR) groups in comparison to healthy control. (**A**) IFN γ: control group: mean 55.26 ± 3.2 and range 43–78; T1D AdV^−^: mean 93.55 ± 2.86 and range 80–121.5; and T1D AdV^+^: mean 115.7 ± 4.57 and range 93–154.4; (**B**) IL-15: control group: mean 14.48 ± 0.73 and range 10.3–21.2; T1D AdV^−^: mean 46.6 ± 1.25 and range 38–55; and T1D AdV^+^: mean 77.64 ± 3.2 and range 63–101; (**C**) adiponectin: control group: mean 14.75 ± 0.36 and range 13.8–19; T1D Adv^−^: mean 17.82 ± 0.417 and range 14–20.5; and T1D Adv^+^: mean 23.08 ± 0.47 and range 15.2–27; and (**D**) microalbuminuria: control group: mean 15.6 ± 0.97 and range 9–24; T1D Adv^−^: mean 24.83 ± 2.09 and range 17.4–29; and T1D Adv^+^: mean 29.1 ± 2.9 and range 21–36.6. Values were considered significantly different at *** *p* < 0.001 versus healthy controls, and ### *p* < 0.001 T1D—AdV^−^ versus T1D—AdV^+^ children. Type 1 diabetes parameters and microalbuminuria.

The levels of FBS and HbA1c% were associated with a significant (*p* < 0.001) increase in T1D diabetic children compared with healthy controls, while C-peptide levels were associated with a significant (*p* < 0.001) decrease in T1D diabetic children compared with healthy controls. The levels of FBS and HbA1c% in the T1D—AdV^−^ and T1D—AdV^+^ groups revealed a highly significant (*p* < 0.001) increase in T1D—AdV^+^ compared with healthy controls and the T1D—AdV^−^ group (Figure 1A,B). Moreover, C-peptide levels were significantly decreased (*p* < 0.001) in the T1D—AdV^+^ group as compared to the T1D—AdV^−^ group (Figure 1C). Regarding microalbuminuria levels in the T1D–AdV^+^ group, the data showed a significant (*p* < 0.01) increase compared to the T1D–AdV^−^ group (Figure 2D).

### 3.2. Assessment of Lipid Profile

**Figure 3:**
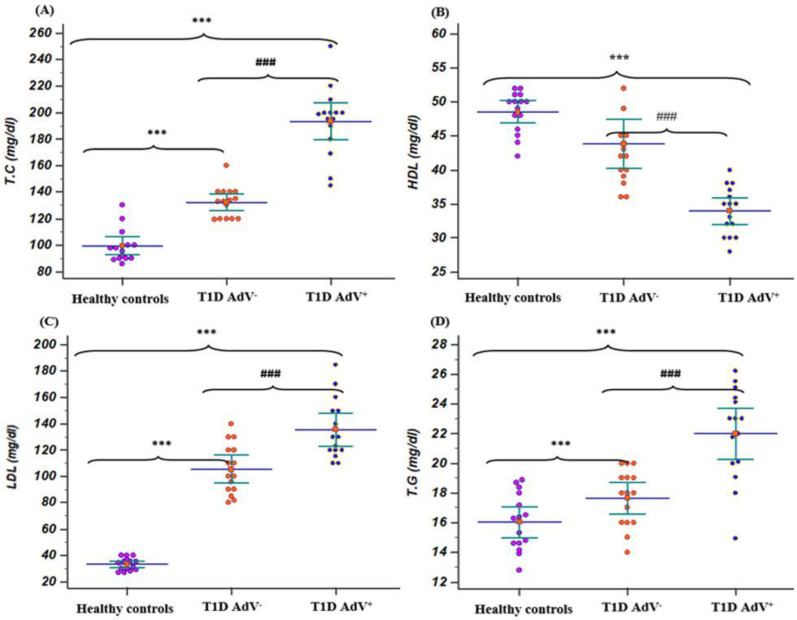
Lipid profile in (T1D—negative AdV (PCR and antibodies) and T1D—AdV positive (PCR) groups in comparison to healthy control. (**A**) Total cholesterol (TC): control group: mean 99.135 ± 3.165 and range 86–130; T1D AdV^−^: mean 132.26 ± 2.93 and range 119–160; and T1D AdV^+^: mean 193.26 ± 6.3 and range 145–250; (**B**) high-density lipoprotein. (HDL): control group: mean 48.53 ± 0.779 and range 42–52; T1D AdV^−^: mean 41.86 ± 1.67 and range 36–54; and T1D AdV^+^: mean 32.6 ± 1.08 and range 28–42; (**C**) low-density lipoprotein(LDL): control group: mean 33.2 ± 1.21 and range of 27–40; T1D AdV^−^: mean 121.73 ± 6.92 and range 80–143; and T1D AdV^+^: mean 143.54 ± 8.17 and range 106–148; and (**D**) triglycerides (T.G): control group: mean 33.2 ± 1.21 and range of 27–40; T1D AdV^−^: mean 121.73 ± 6.92 and range 80–143; and T1D AdV^+^: mean 143.54 ± 8.17 and range 106–185. Values were considered significantly different at *** *p* < 0.001 versus healthy controls, and ### *p* < 0.001 T1D—AdV^−^ versus T1D—AdV^+^ children. T.C.

Total cholesterol, LDL-c, and triglycerides levels were markedly (*p* < 0.001) increased in the T1D–AdV^+^ group compared to the T1D–AdV^−^ group (Figure 3A,C,D). Additionally, HDL-c levels were noticeably (*p* < 0.001) decreased in the T1D–AdV^+^ group compared to the T1D–AdV^−^ group (Figure 3B).

### 3.3. Adenovirus Detection

Positive PCR for adenoviruses was evidenced in 20% of the children with T1D (16 out of 80) and found to be significantly (*p* < 0.05) higher than that detected in healthy control children (1 out of 40, 2.5%) (Table 1). Moreover, when compared between the newly and previously diagnosed T1D groups, the results showed that a positive adenovirus PCR was more frequently detected in children with newly diagnosed T1D (10 out of 30, 33.4%) in comparison to previously diagnosed children (6 out of 50, 12%) (*p* < 0.05) (Table 1).

The seroprevalence of anti-adenoviruses IgG revealed the presence of specific anti-adenovirus IgG in 12.5% (5 out of 40) of healthy controls in comparison to 40% (32 out of 80) of diabetic children (*p* < 0.05; Table 1). Anti-Adenovirus IgG revealed 46.7% (14 out of 30) were positive in the newly diagnosed group, but the previously diagnosed T1D group exhibited 36% (18 out of 50) when compared to healthy controls (*p* < 0.05; Table 1). No significant correlation was found between age or BMI with either adenovirus active infection as evidenced by a positive PCR reaction or seroconversion (Table 1). Among the T1D children, 2/11 (age 2–5 Mo), 2/12 (age 6–10 Mo), 11/52 (age 11–15 Mo), 2/5 (age 16–20 Mo) were found to be positive.

### 3.4. Cytokines Levels

Regarding T1D—AdV^+^ and T1D—AdV^−^ groups, IFN-γ and IL-15 levels revealed a significant increase when compared to healthy controls (*p* < 0.001; Figure 2A,B). While adiponectin was upregulated significantly (*p* < 0.001) in the T1D—AdV^+^ group compared to the T1D–AdV^−^ and healthy control groups (Figure 2C).

### 3.5. Correlations Results

Considerable correlation was found between the presence of a positive adenovirus PCR result and total cholesterol (*r* = 0.196, *p* < 0.032). LDL was also found to be correlated with positive adenovirus PCR and the presence of adenovirus IgG (*r* = 0.21, *p* < 0.021) and (*r* = 0.183, *p* < 0.045) (Table 2). Both INFγ and IL-15 exhibited a highly significant correlation with adiponectin and C peptide (Table 3). A highly significant negative correlation was detected for adiponectin and C peptide with IL-15, INFγ, triglycerides, adiponectin, C peptide, HbA1C, microalbumin, creatinine LDL, HDL, and TC (Table 2 and Table 3).

## 4. Discussion

T1D is a chronic inflammatory disease the etiology of which is not fully understood. Β-cell loss in T1D results from a multifactorial process, involving host genes, environmental factors, and an autoimmune response. Viral infections are among the incriminated causes that could lead to T1D as they have been isolated from the pancreas of T1D patients [16].

The adopted nested PCR protocol for adenovirus detection was proved to successfully detect 47 adenovirus serotypes (Ad1-Ad 47) that belong to the subgenera A-F [15]. In the current study, the prevalence of adenovirus DNA was 20% positive in T1D children compared to 2.5% positive in healthy control children. The current finding is supported by the recent detection of adenovirus HAdV-F and HAdV-C in young children; however, HAdC infections were only found to be associated with islet autoimmunity [4]. The current finding augments the possibility that adenovirus infection could be among the triggering factors of T1D. It is known that viral DNA detection in the serum denotes an active infection, which might be sufficient for the induction of autoimmunity before the development of anti-viral IgG [17]. Thus, adenovirus infection may exacerbate β-cell destruction and subsequently decrease the secretion of insulin. This was also confirmed by the significant increased level of HbA1c, INFγ, IL-15, and adiponectin in the T1D–AdV^+^ group.

In the current study, anti-adenovirus IgG was detected in 40% of diabetic children compared to 12.5% of healthy controls. In a previous study, adenovirus-seropositive T1D patients showed high antibody titers to glutamic acid decarboxylase. Such a finding suggests that adenovirus infections may trigger autoimmunity to pancreatic β cells [18]. Adenovirus infection of children, younger than 6 months old, is less likely to induce T1D via islet autoimmune responsiveness [4]. Although we did find considerable numbers of T1D in 2-5-month-old children, the majority of cases were detected in 11-15-month-old children.

Moreover, we found that the prevalence of adenovirus DNA was 33.4% in children with newly diagnosed T1D and 12% in previously diagnosed children. While anti-adenovirus IgG was found in 46.7% of the newly diagnosed T1D group and 36% in the previously diagnosed T1D group. The detection of IgG in the presence of a positive adenovirus PCR may indicate infection with different serotypes or a state of persistent adenovirus infection. The later strategy was recorded in HAdV-C infections as the virus persisted for long periods [19]. Cell destruction by virus-specific cytotoxic T cells with subsequent tissue destruction and the release of self-antigens could result in the activation of autoreactive T cells with the development of an autoimmune response [20]. It is apt to mention that adenoviruses have been speculated to invade β cells and destroy β cells with the probable development of autoimmunity as in the case of coxsackieviruses [4].

In contrast, a protective rather than destructive role of adenoviruses in T1D has been speculated [4]. Both adenovirus and coxsackie B virus bind to the same receptor, *CXADR*, so possible interference does exist between these two viruses. Early HAdV-C infections, before the age of 6 months, was associated with a low risk of islet autoimmunity, and this was assumed to be a mechanism for protection by HAdV-C against infection by coxsackie B virus [4]. However, a significant increase in the existence of the positive adenovirus in T1D children in comparison to children with HAdV-ve T1D suggests the potential destructive rather than protective role of adenoviruses.

Cytokines and other inflammatory mediators promote the Th1 response. They increase the expression of MHC and adhesion molecules in antigen-presenting cells [21]. In T1D-associated autoimmunity, the Th1 phenotype responds by producing IFN-γ. This fact was clearly observed in our study as IFN-γ production was significantly upregulated in both the T1D—AdV children’s groups in comparison to healthy controls. In addition, HbA1c% was found to be positively correlated with IFN-γ. IFN–γ was reported to be responsible for the activation of natural killer (NKs) cells, which, in turn, are responsible for pancreatic β-cell apoptosis [22]. TNF-α and IFN-γ are involved in pancreatic β-cell apoptosis through the activation of calcium channels, which, in turn, induce caspase activation [23].

In addition, IL-15 production was significantly upregulated and was highly positively correlated with HbA1c% in the T1D–AdV^+^ compared to T1D–AdV^−^ children group. This finding is in agreement with a previous study that demonstrated an increase in the levels of IL-15 or IL-15Rα in the serum and/or in the islets of T1D patients [24]. IL-15 induces proliferation of CD4+ and CD8+ T cells and secretion of INF-γ and TNF-α by NK cells. The increasing levels of IFN-γ and IL-15 in the T1D–AdV^+^ T1D children’s groups were seen where children with islet autoimmunity demonstrate higher levels of multiple cytokines. Our study revealed that IL-15 level was highly increased in the T1D–AdV^+^ group, most probably due to viral infection, as in the case of enterovirus infection and coxsackievirus B4 [22]. In our study, adiponectin was negatively associated with HbA1c and BMI. These findings are in agreement with the evidence that visceral fat accumulation, BMI, triglyceride glucose, and insulin levels are inversely correlated with the levels of adiponectin [25]. Infections with human adenovirus serotype 5, 36, or 37 have been reported to be associated with increased BMI and obesity [26]. Interestingly, infections with adenoviruses are reported to be linked with adiposity in both experimental animal models and human studies [27]. On the contrary, a significant increase in serum adiponectin levels in the T1D–AdV^+^ compared with T1D–AdV^−^ and healthy control group was found in our study. An increase in the concentration of adiponectin was detected in patients with poorly controlled T1D [28]. In our study, adiponectin was positively correlated with C-peptide. The predicted adiponectin concentrations at 1 month, 6 months, and 12 months of T1D follow-up, respectively, were associated with distinct progression patterns of T1D after the establishment of diagnosis [29]. High adiponectin levels were found to be correlated with poor diabetes control, while dyslipidemia, lower concentrations of adiponectin, and higher levels of inflammatory and endothelial biomarkers were correlated with obesity [29]. Natural AdV36 infections were correlated with obesity and metabolic disorders in children and adults of different ethnic groups and populations in different countries [30,31,32,33]. Approximately 30% of obese and 11% of nonobese humans had neutralizing antibodies to HAdV-36, which was associated with reductions in serum cholesterol and triglycerides [33]. AdV-36 was strongly associated with lipid disorders in Korean schoolchildren [31].

Higher adiponectin levels in TID have been reported to be associated with the onset and progression of microalbuminuria [34]. A possible explanation could be that adiponectin can change the integrity of endothelial junctions and induce NO production, thus affecting hyperfiltration [35]. In our study, microalbuminuria levels were noticeably increased in T1D–AdV^+^ children compared with T1D—AdV^−^ or healthy children. Notably, structural changes associated with diabetic kidney disease may develop as early as 1.5–5 years after the onset of T1D [36].

## 5. Conclusions

In conclusion, adenovirus infection may have a role in the development and progression of T1D. IFN-γ, IL-15, and adiponectin were associated with adenoviruses-induced T1D.

## 6. Study Limitation

The main limitation of the present study is the small number of included subjects. Moreover, positive adenovirus samples needed to be sequenced to identify their serotype; however, this may have been related to the abundance, which is inappropriate for direct sequencing. This study also lacked auto-antibody detection, such as islet cell cytoplasmic auto-antibodies (ICA), auto-antibodies targeting insulin, islet antigen-2 protein, and glutamic acid decarboxylase.

## Figures and Tables

**Table 1:** Demographic data of healthy controls, recently diagnosed T1DM, and previously diagnosed T1D children expressed as numbers and percentages.

		Group				Sig.	Adenovirus PCR		Sig.	Adenovirus IgG		Sig.
		Control N:40		Previously Diagnosed with T1D, N:50	Recently Diagnosed with T1D, N:30		Negative	Positive		Negative	Positive	
Gender	Male	No	25	27	15	0.548	59	8	0.618	49	18	0.290
		%	62.5	54.0	50.0		57.3	47.1		59.0	48.6	
	Female	No	15	23	15		44	9		34	19	
		%	37.5	46.0	50.0		42.7	52.9		41.0	51.4	
Age	2–5 Mo	No	5	1	10	0.001	14	2	0.15	11	5	0.962
		%	12.5	2.0	33.3		13.6	11.8		13.3	13.5	
	6–10 Mo	No	23	6	6		33	2		24	11	
		%	57.5	12.0	20.0		32.0	11.8		28.9	29.7	
	11–15 Mo	No	12	39	13		53	11		44	20	
		%	30.0	78.0	43.3		51.5	64.7		53.0	54.1	
	16–20 Mo	No	0	4	1		3	2		4	1	
		%	0.0	8.0	3.3		2.9	11.8		4.8	2.7	
BMI	Severely underweight	No	9	2	8	0.001	15	4	0.736	13	6	0.363
		%	22.5	4.0	26.7		14.6	23.5		15.7	16.2	
	Underweight	No	26	12	6		39	5		32	12	
		%	65.0	24.0	20.0		37.9	29.4		38.6	32.4	
	Normal	No	4	23	12		34	5		23	16	
		%	10.0	46.0	40.0		33.0	29.4		27.7	43.2	
	Overweight	No	0	12	3		12	3		12	3	
		%	0.0	24.0	10.0		11.7	17.6		14.5	8.1	
	Obese	No	1	1	1		3	0		3	0	
		%	2.5	2.0	3.3		2.9	0.0		3.6	0.0	

**Table 2:** Correlation between the existence of adenovirus DNA and anti-IgG and biochemical parameters.

		Adenovirus PCR	Adenovirus IgG	TG	HbA1C	Microalbumin	Creatinine	LDL	HDL	TC
Adenovirus PCR	R	1.000	0.143	−0.054	0.151	0.012	0.100	0.210 *	0.011	0.196 *
	P	.	0.120	0.556	0.101	0.898	0.278	0.021	0.904	0.032
Adenovirus IgG	R		1.000	0.043	0.173	0.137	0.113	0.183 *	0.058	0.163
	P		.	0.639	0.059	0.135	0.218	0.045	0.527	0.075
TG	R			1.000	0.333 **	0.213 *	0.291 **	0.526 **	0.138	0.573 **
	P			.	0.001	0.020	0.001	0.001	0.133	0.001
HbA1C	R				1.000	0.589 **	0.501 **	0.641 **	0.225 *	0.626 **
	P				.	0.001	0.001	0.001	0.014	0.001
Microalbumin	R					1.000	0.493 **	0.622 **	-0.002	0.579 **
	P					.	0.001	0.001	0.982	0.001
Creatinine	R							0.554 **	0.073	0.543 **
	P							0.001	0.429	0.001
LDL	R								0.116	0.866 **
	P								0.206	0.001
HDL	R									0.192 *
	P									0.035
TC	R									
	P									

Biochemical parameters in newly diagnosed T1D, previously diagnosed T1D, and control groups were correlated together using bivariate Spearman correlation to determine whether there was possible link between different parameters with regard to exposure to adenovirus infection. BMI: body mass index; TG: triglycerides; TC: total cholesterol; **. Correlation is significant at the 0.01 level (2-tailed). *. Correlation is significant at the 0.05 level (2-tailed).

**Table 3:** Correlation between the existence of adenovirus DNA and anti-IgG and the level of IL15,I NFγ, adiponectin, and C peptide.

		Adenovirus PCR	Adenovirus IgG	BMI	IL15	INFγ	Adiponectin	C Peptide
Adenovirus PCR	R	1	0.143	−0.026	0.181	−0.052	−0.077	−0.080
	P	.	0.12	0.778	0.059	0.575	0.425	0.383
Adenovirus IgG	R		1	−0.012	0.181	0.059	−0.190 *	−0.177
	P		.	0.894	0.058	0.523	0.046	0.053
BMI	R				0.259 **	0.329 **	−0.253 **	−0.433 **
	P				0.006	0.001	0.008	0.001
IL15	R					0.186	−0.572 **	−0.576 **
	P					0.052	0.001	0.001
INFγ	R					1	−0.267 **	−0.469 **
	P					.	0.005	0.001
Adiponectin	R							0.611 **
	P							0.001
C peptide	R							1
	P							.

The levels of IL15, INFγ, adiponectin, and C peptide in the newly diagnosed T1D, previously diagnosed T1D, and control groups were correlated using bivariate Spearman correlation together to determine the whether there was possible link between different parameters with regard to exposure to adenovirus infection. **. Correlation is significant at the 0.01 level (2-tailed), *. Correlation is significant at the 0.05 level (2-tailed).

## Data Availability

Data is contained within the article.
